# Glioblastoma stem cells as carriers of tumour memory: a strategy for personalised immunotherapy using tumour-infiltrating lymphocytes

**DOI:** 10.3389/fimmu.2026.1880908

**Published:** 2026-07-22

**Authors:** Igor Bryukhovetskiy, Oleg Pak, Alexander Polevshchikov

**Affiliations:** 1 Far Eastern Federal University Hospital, Vladivostok, Russia; 2 Laboratory of General Immunology, Institute of Experimental Medicine, Saint-Petersburg, Russia

**Keywords:** cancer stem cells, glioblastoma, microenvironment, T memory stem cells (TSCM), tumour-infiltrating lymphocytes (TILs)

## Abstract

Glioblastoma (GB) remains one of the most aggressive brain tumours, with a median survival of 15 months, largely due to resistance towards available anti-cancer therapies, including cutting-edge immunotherapy. Growing evidence indicates involvement of cancer stem cells (CSCs) in escalating resistance against existing treatment modalities due to their phenotypic plasticity, elevated expression of drug-resistance pumps, and immunomodulatory behaviour. Such oncogenic consequences are regulated by several epigenetic reprogramming events that are pivotal in retaining adaptive traits associated with CSC-mediated therapy resistance and subsequent oncogenic progression. In this review, we consider such epigenetic consequences as “tumour memory” and try to shed light on the therapeutic vulnerabilities by targeting CSCs — the “carriers of tumour memory” — via immune interventions in GB. Interestingly, immune-based anti-cancer therapies are coming to the forefront of cancer research, where T lymphocytes are immunologically boosted to attack tumour cells. However, in reality, several constraints burden such procedures. Contextually, the GB microenvironment, dominated by bone marrow-derived cells, reprograms infiltrating immune cells into suppressor phenotypes, creating a “cold” immune landscape. The only zone where functionally active lymphocytes are preserved is the invasive margin of the tumour, where they undergo exhaustion along the TPE → TEX axis but retain proliferative potential. On this basis, we introduce the concept of “invasive margin lymphocytes” (IMLs), of which stem-like memory T cells (TSCM) are indispensable for long-term immunological protection, as they have unique proliferative potential and ability for long-term persistence. Incidentally, such TSCMs have striking similarities with CSCs, as both cell types employ evolutionarily conserved mechanisms of the WNT/β-catenin signalling pathway, hierarchical organisation, and DNA repair systems. Therefore, understanding CSC–TSCM bidirectional cross-talk could provide a heuristic basis for developing personalised TIL-based therapy aimed at suppressing the hierarchically organised CSC population and overcoming CSC-guided immunotherapy resistance. Drawing conceptual parallels between GB CSCs and TSCM, here in this review, we propose a three-stage therapeutic strategy: precision cytoreduction of the invasive margin, “warming up” the microenvironment using cancer vaccines and pharmacological agents, as well as adoptive transfer of TILs derived from the IML pool to ensure durable disease control.

## Highlights

- Glioblastoma cancer stem cells (CSCs), similarly to T memory stem cells (TSCM), share common evolutionarily conserved mechanisms (WNT/β-catenin signalling, DNA repair, hierarchical organisation), which may indicate the existence of “tumour memory”. However, the functional outcomes of these pathways differ substantially between the two cell types, and the analogy is heuristic rather than mechanistic.
- ”Tumour memory” (defined as the epigenetically fixed ability of CSCs to retain adaptive traits) protects the neoplasm from treatment. This concept requires experimental validation through *in vitro* models of acquired resistance, single-cell chromatin analysis and lineage tracing studies.
- The glioblastoma microenvironment is a highly organised ecosystem that recruits BMDCs and forcibly reprograms them into suppressor phenotypes (M2, N2, Treg) that support CSCs.
- Effective anti-tumour lymphocytes may survive only at the tumour periphery, where they undergo exhaustion along the TPE → TEX axis and form tertiary lymphoid structures. The existence of IMLs is a working hypothesis requiring spatial transcriptomics and multiplex immunofluorescence validation.
- A three-stage engineering strategy for managing the CSC microenvironment is proposed: 1) precision cytoreduction of the invasive margin; 2) “warming up” the TME using pharmaceuticals and cancer vaccines; 3) adoptive TIL therapy (from the IML pool). We acknowledge significant clinical challenges (production time, BBB penetration, IL-2 toxicity) that require further investigation.

## Introduction

Glioblastoma (GB) remains one of the most aggressive and therapeutically resistant tumours of the central nervous system. Despite the implementation of the current standard of care, including maximally safe surgical resection, radiotherapy and temozolomide chemotherapy, median overall survival does not exceed 15 months, and five-year survival is less than 5% (1, 2). Tumour recurrence after initial response to therapy remains virtually inevitable, indicating the existence of fundamental resistance mechanisms that are not eliminated by current treatment modalities.

A key role in the formation of therapeutic resistance and recurrence of GB is played by cancer stem cells (CSCs) — a subpopulation of cells possessing self-renewal capacity, hierarchical organisation and high tumourigenicity (3, 4). These cells are today considered the main drivers of tumour progression, as they are capable of surviving cytotoxic exposure by activating DNA repair mechanisms, reducing the degree of differentiation and adapting to extreme microenvironmental conditions — hypoxia, lactate acidosis and immune pressure (5, 6). However, the ability of CSCs to survive and transmit stable properties to progeny goes beyond simple “resistance” and may be characterised as **“**tumour memory**”** — an epigenetically fixed ability to preserve and transmit adaptive traits acquired under pressure from therapy and the microenvironment (7, 8). We emphasise that this concept is a working hypothesis requiring experimental validation, as detailed in Section 5.3.

Remarkably, cancer stem cells demonstrate functional similarity to T memory stem cells (TSCM) — the most long-lived subpopulation of T cells responsible for the formation and maintenance of immunological memory (9, 10). Both cell types employ common evolutionarily conserved mechanisms: the WNT/β-catenin signalling pathway for maintaining self-renewal, DNA repair systems for preserving genomic integrity, hierarchical population organisation and the ability for dynamic chromatin modulation in response to external signals (11–13). However, as we critically examine in Section 5.2 (**Table 1**), the functional outcomes of these pathways differ substantially between the two cell types. WNT/β-catenin signalling in TSCM primarily maintains quiescence and self-renewal through TCF1, whereas in GB CSCs it often stimulates proliferation, invasion and therapy resistance. DNA repair in TSCM is essential for the formation of “epigenetic scars” underlying memory maintenance, whereas in CSCs it serves as a survival tool against genotoxic stress. Thus, the similarity is heuristic rather than mechanistic, and we use it as an instrument for generating hypotheses rather than as an established biological fact.

**Table 1:** Comparison of signalling and DNA repair mechanisms in glioblastoma CSCs and TSCM.

Mechanism	Glioblastoma CSCs	TSCM	Functional differences
WNT/β-catenin	Activation via TGFβ; stimulates proliferation, invasion, expression of resistance genes (ABC transporters, Survivin)	Activation via Wnt ligands from niche; maintains quiescence and self-renewal through TCF1	In TSCM — maintenance of stemness and prevention of exhaustion; in CSCs — driver of aggressive phenotype
DNA repair (NHEJ)	Survival under genotoxic stress; activation of DNA-PK, Ku70/Ku80, XRCC4	Formation of “epigenetic scars”; involvement in V(D)J recombination	In CSCs — survival tool; in TSCM — basis of long-term memory
DNA repair (BER)	Removal of oxidative damage; activation of PARP1, APE1, DNA polymerase β	Maintenance of genomic stability during homeostatic proliferation	In CSCs — protection from oxidative stress in hypoxia; in TSCM — maintenance of genomic integrity
Chromatin modifiers	PRDM9 (H3K4me3), EZH2 (H3K27me3), HDACs	PRDM9, EZH2, HDACs (similar set, different target genes)	Shared enzymes but different target gene repertoires; in CSCs — maintenance of resistance; in TSCM — maintenance of memory
Hierarchical organisation	Hierarchy: CSC → progenitor cells → differentiated cells	Hierarchy: TSCM → TCM → TEM → TEMRA	In CSCs — basis of heterogeneity and resistance; in TSCM — basis of long-term immune protection
Microenvironment dependence	Dependence on niche (hypoxia, TGFβ, signals from BMDCs)	Dependence on niche (IL-7, IL-15, signals from dendritic cells)	In CSCs — maintenance of suppressive TME; in TSCM — maintenance of homeostasis

Key conclusion: Although CSCs and TSCM share common signalling pathways and enzymatic systems, the functional outcomes of these pathways differ substantially. WNT/β-catenin in TSCM maintains quiescence and prevents exhaustion, whereas in CSCs it stimulates proliferation and resistance. DNA repair in TSCM serves as the basis for “epigenetic scars” and long-term memory, whereas in CSCs it is a survival tool under genotoxic stress. Thus, the analogy is heuristic rather than mechanistic and requires cautious interpretation.

The GB microenvironment creates serious obstacles to the implementation of anti-tumour immunity. In the central zones of the tumour, where hypoxia and lactate acidosis are pronounced, a “cold” immune landscape is formed with a predominance of bone marrow-derived cells (BMDCs) reprogrammed into immunosuppressive phenotypes — M2-macrophages, N2-neutrophils and regulatory T cells (Treg) (14, 15). These cells produce transforming growth factor β (TGFβ), which not only suppresses the effector functions of lymphocytes but also induces their exhaustion along the TPE → TEX model, characterised by progressive loss of proliferative potential and cytotoxic activity (16, 17).

In this context, a key question arises: do lymphocytes capable of an anti-tumour response persist in the tumour? The literature indicates that the only zone where functionally active lymphocytes may be preserved is the invasive margin of the tumour — the transitional area between the neoplasm and healthy brain tissue (18). It is here, under conditions of higher partial oxygen pressure and less pronounced immunosuppressive influence, that a subpopulation of lymphocytes may persist which, although undergoing exhaustion, may not completely lose its proliferative potential. We hypothesise the existence of this therapeutically significant subpopulation and propose to designate it as **“**invasive margin lymphocytes” (IMLs). We explicitly acknowledge that this is a working hypothesis requiring validation through spatial transcriptomics, multiplex immunofluorescence and single-cell sequencing (see Section 6.4).

Thus, the central hypothesis of this review is as follows: the convergent similarity between glioblastoma CSCs and T memory stem cells may serve as a heuristic basis for developing an immunotherapy strategy aimed at suppressing “tumour memory” through the adoptive transfer of TILs derived from the putative subpopulation of invasive margin lymphocytes. This approach allows TIL therapy to be viewed not merely as a method of enhancing anti-tumour immunity but as a tool for the targeted management of the hierarchically organised CSC population — the carriers of tumour memory.

The aim of this review is to analyse current data on the role of TILs in GB pathogenesis, identify conceptual parallels between CSCs and TSCM, and propose a novel three-stage therapeutic strategy integrating active immunotherapy with the adoptive transfer of TILs derived from the IML pool. We do not postulate a direct ontological correspondence but use the identified similarity as a heuristic tool for searching for new therapeutic targets and developing personalised approaches to GB treatment. We also critically evaluate the clinical feasibility of the proposed strategy and identify key knowledge gaps requiring future investigation.

## Current treatment modalities for GB and their limitations

The current standard of care for newly diagnosed GB includes maximally safe surgical resection followed by radiotherapy and temozolomide (TMZ) chemotherapy according to the EORTC-26981/22981 protocol (18). This strategy, while remaining the cornerstone of therapy, increases median overall survival to 15 months; however, two-year survival does not exceed 15%, and five-year survival — 2% (19, 20). In recurrence, repeat surgery is performed in only 30% of patients, and a third operation in less than 10% (21). These figures convincingly demonstrate that current approaches do not allow achieving durable disease control.

Surgical treatment, despite the development of intraoperative navigation, fluorescence visualisation with 5-aminolevulinic acid and awake neurosurgery, faces fundamental limitations. The invasive growth of GB, in which tumour cells migrate from the primary focus over a distance of up to 7 cm or more, makes radical elimination of all tumour cells by surgery impossible. Furthermore, tumour localisation in functionally significant brain areas (central gyri, speech centres, brainstem) significantly limits the volume of resection (22, 23). Even with the most radical surgery, a significant proportion of tumour cells, including CSCs, remain in the adjacent brain tissue, forming the basis for future recurrence.

Radiotherapy (total dose 60 Gy, 2 Gy daily) and chemotherapy with TMZ are aimed at eliminating residual tumour cells after surgery. However, their efficacy is limited by several factors. First, CSCs exhibit pronounced radio- and chemoresistance: in response to genotoxic damage, they reduce the degree of differentiation and activate DNA repair systems, including homologous recombination and non-homologous end joining, allowing them to evade death (24, 25). Second, the blood-brain barrier (BBB), although disrupted in the central zones of the tumour, remains a significant obstacle to the penetration of chemotherapeutic agents, especially in areas with the most intense tumour cell infiltration (26–28). The concentration of TMZ in brain tissue is only 10–30% of that in plasma, and dose escalation above 200 mg/m² is limited by myelosuppression and systemic toxicity (29, 30).

Importantly, standard chemotherapy, while effectively eliminating the differentiated fraction of tumour cells, can paradoxically selectively enrich the CSC microenvironment, inducing their transition to a more primitive, resistant state (31). This phenomenon, known as the formation of “drug-tolerant persister cells,” is associated with reversible epigenetic rearrangements, including activation of the histone methyltransferase PRDM9 and adaptive chromatin remodelling (32, 33). Thus, standard therapy, while remaining necessary, creates an additional challenge — it promotes the maintenance of the hierarchically organised CSC population, which may act as a carrier of “tumour memory.”

Alternative strategies, including the use of angiogenesis inhibitors (bevacizumab), combinations of cytostatics with bevacizumab, intrathecal administration of drugs via an Ommaya reservoir, or the use of Tumour-Treating Fields, expand the therapeutic arsenal but do not solve the problem of fundamental CSC resistance (34–36). Moreover, these approaches are often associated with additional toxicity and do not demonstrate convincing increases in overall survival.

Thus, current GB treatments face a common problem: they effectively target the differentiated fraction of tumour cells but do not eliminate the CSC population, which, due to its plasticity, DNA repair systems and ability to interact with the microenvironment, may retain “memory” of past treatment and ensure tumour recurrence. This makes CSCs the most attractive but at the same time the most challenging target for targeted therapy and immunotherapy. Understanding the biology of CSCs and the mechanisms of their interaction with the microenvironment becomes a prerequisite for the development of fundamentally new therapeutic approaches capable of overcoming therapeutic resistance and ensuring durable disease control.

## Glioblastoma stem cells and tumour memory

### CSCs as the cellular basis of resistance and “tumour memory”

The concept of CSCs was introduced into scientific discourse more than 20 years ago to denote a subpopulation of tumour cells with high tumourigenicity and the ability to reproduce an authentic tumour when implanted into experimental animals in small numbers (37). Later, these cells became associated with therapeutic resistance and recurrence (38). It is now clear that the elimination of CSCs is a priority goal of all existing targeted therapy approaches. However, despite intensive research, a single immunophenotypic marker of CSCs has not been identified: tumour cells expressing CD133, CD15, SOX2, A2B5 and other markers demonstrate significant heterogeneity, and tumourigenicity is not associated with any single immunophenotypic subpopulation (39–43). This indicates that the phenomenon of “stemness” in GB is primarily a functional and highly dynamic property, rather than a rigidly fixed cellular phenotype. In other words, the identification of CSCs is possible not by a set of formal phenotypic or genetic characteristics, but by a set of functional properties.

The key functional characteristic of CSCs is plasticity — the ability to adapt to the destructive effects of external factors: hypoxia, lactate acidosis, radiotherapy and chemotherapy (44). Plasticity increases as the degree of CC differentiation decreases, is accompanied by genome demethylation and reaches a maximum in CSCs (25). It is this ability that allows CSCs to survive in extreme microenvironmental conditions, activating a wide arsenal of DNA single- and double-strand break repair mechanisms, including homologous recombination and non-homologous end joining. However, plasticity is not limited to survival: CSCs are capable of forming a hierarchical structure, integrating into the system of local intercellular interactions and adapting to immune pressure, which makes them not just “resistant” cells but organisers of the tumour ecosystem.

A critically important property of CSCs is their ability to preserve and transmit to progeny — predominantly during asymmetric division — the epigenetic and transcriptional status associated with therapy resistance, hypoxic survival and collaboration with the microenvironment (45, 46). This ability underlies the phenomenon we hypothesise as “tumour memory.”

We define “tumour memory” as a dynamically maintained, epigenetically encoded ability of cancer stem cells to preserve and transmit to progeny (predominantly during asymmetric division) a transcriptional programme associated with survival under conditions of therapy, hypoxia and immune pressure. In contrast to “stemness” (the ability for self-renewal and multipotency), “memory” focuses on the retention over time of specific adaptive traits. In contrast to “plasticity” (the ability to change phenotype), “memory” implies that after returning to the original conditions, the cell retains a “trace” of the previous exposure — for example, maintains open chromatin at resistance gene loci (47). In contrast to “acquired resistance” (genetically fixed stability), “memory” implies reversible, non-genetic mechanisms. Resistance to therapy is a particular case where memory manifests as epigenetically fixed tolerance to temozolomide or radiation.

Tumour memory is not a fixed state but a dynamically maintained property that is modulated by niche signals and can reversibly change upon TME alteration (32, 33). This dynamism has direct clinical significance: standard therapy, especially when carried out without consideration of molecular-genomic characteristics and individual tumour sensitivity, can paradoxically selectively enrich the CSC microenvironment and induce their transition to a more primitive state (31). This does not mean that temozolomide or other chemotherapeutics “exacerbate the disease” — they remain the cornerstone of treatment and have proven survival benefits. However, it reveals an additional challenge: the need to combine standard therapy with methods targeting CSCs and their memory to prevent relapse.

The immunomodulatory properties of CSCs also play an important role in maintaining tumour memory. CSCs actively interact with the microenvironment, secreting chemokines (CXCL12, CXCL10, CXCL9) that attract bone marrow-derived cells (BMDCs) and reprogram them into suppressor phenotypes (48–50). Myeloid suppressor cells, M2-polarised macrophages and N2-neutrophils induced by CSCs produce TGFβ, IL-10 and reactive oxygen species, creating an immunosuppressive landscape that protects CSCs from elimination (51, 52). Thus, CSCs act not just as passive “survivors” but as active architects of the microenvironment, maintaining their own niche and memory through a complex network of paracrine signals.

### Molecular-epigenetic mechanisms of tumour memory: similarities and differences with TSCM

**Figure 1:**
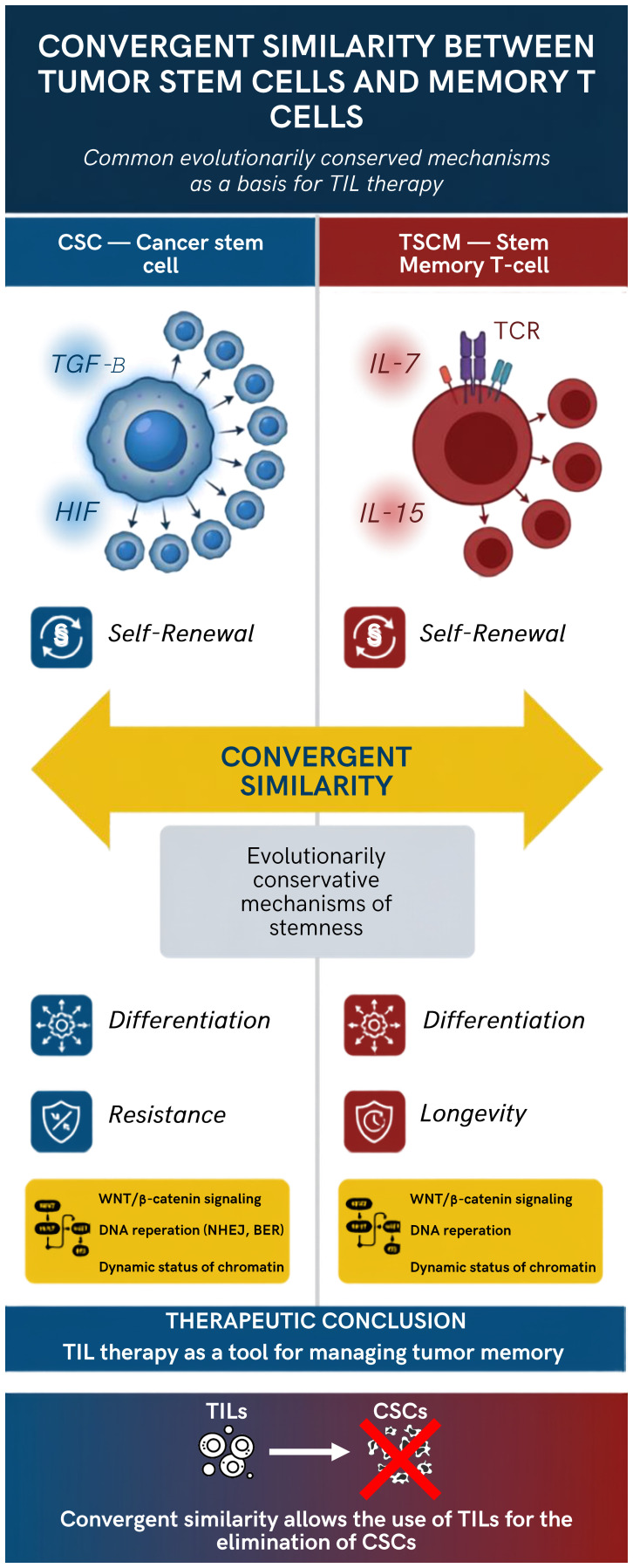
Convergent similarity between glioblastoma cancer stem cells (CSCs) and T memory stem cells (TSCM). The image illustrates the conceptual parallelism between CSCs and TSCM, which serves as the heuristic foundation for the proposed TIL-based therapeutic strategy. The left panel depicts glioblastoma CSCs characterised by self-renewal capacity, hierarchical organisation, WNT/β-catenin signalling, DNA repair systems and dynamic chromatin status, which collectively underpin “tumour memory”. The right panel depicts T memory stem cells (TSCM) characterised by self-renewal, multipotency, WNT/β-catenin signalling, DNA repair and dynamic chromatin status, which together form the basis of “immunological memory”. The central bidirectional arrow highlights the convergent similarity between the two cell types, emphasising their shared evolutionarily conserved mechanisms of stemness, including self-renewal, differentiation potential, resistance and longevity. The lower panel illustrates the therapeutic conclusion: TILs (tumour-infiltrating lymphocytes) can be directed against CSCs, and the convergent similarity between CSCs and TSCM allows the use of TILs for the elimination of CSCs, thereby managing tumour memory. CSC, cancer stem cell; TSCM, T memory stem cell; TGF-β, transforming growth factor beta; HIF, hypoxia-inducible factor; TCR, T-cell receptor; IL, interleukin; TIL, tumour-infiltrating lymphocyte.

At the molecular level, tumour memory is implemented through a complex of interconnected epigenetic and signalling mechanisms that ensure long-term preservation of adaptive traits. However, while CSCs and TSCM share certain molecular pathways, the functional outcomes of these pathways differ substantially. We critically examine these differences in **Table 1**, **Figure 1**.

Central to the maintenance of tumour memory is epigenetic reprogramming. GB cells are characterised by significant diversity of epigenetic landscapes, which are integrated into four main phenotypic states characteristic of CSCs: neural- and oligodendroglial-progenitor-like, astrocyte-like and mesenchymal-like (53, 54). The formation of these states results from strong “pressure” from the microenvironment, but while adapting to this influence, the cells do not lose their special status — they retain memory, which manifests as maintenance of open chromatin at loci of genes responsible for survival and resistance (47).

A key epigenetic mechanism is adaptive chromatin remodelling (33). In response to genotoxic damage or hypoxia, CSCs activate histone modifiers that rearrange chromatin architecture, making accessible genes associated with survival, DNA repair and metabolic adaptation. An important player in this process is the histone methyltransferase PRDM9, which provides metabolic reprogramming and promotes the formation of a population of “drug-tolerant persister cells” — cells that survive chemotherapy through reversible, non-genetic mechanisms (32).

The WNT/β-catenin signalling pathway acts as a key integrator of microenvironmental signals and the epigenetic status of CSCs. Activation of WNT signalling maintains “stemness” and memory, preventing differentiation and apoptosis (55, 56). However, the functional role of WNT/β-catenin differs between CSCs and TSCM. In TSCM, WNT/β-catenin primarily maintains quiescence and self-renewal through TCF1; in GB CSCs, it often stimulates proliferation, invasion and therapy resistance, with potentially different target gene repertoires. As will be discussed in detail in the next section, TGFβ produced in the GB microenvironment activates AKT and p38 MAPK kinases, leading to inhibition of GSK-3β and accumulation of β-catenin in the cytoplasm and nucleus (57, 58). Translocated to the nucleus, β-catenin binds to transcription factors of the TCF/LEF family, initiating expression of genes ensuring CSC survival and self-renewal.

Genomic heterogeneity and also contribute to tumour memory. Active DNA repair characteristic of CSCs not only ensures survival but also increases the genetic diversity of tumour cells (25). This process creates subclonal heterogeneity, allowing the tumour to “select” the most adapted clones under therapeutic pressure. CSC heterogeneity, in turn, enhances their metastatic potential and invasive capacity, as different subpopulations may use different strategies of migration and survival.

### Knowledge gaps and validation requirements for the “tumour memory” concept

The concept of “tumour memory” proposed in this review is a working hypothesis requiring experimental validation. To confirm or refute this hypothesis, the following studies are necessary:

1. *In vitro* models of acquired resistance. Culture of glioblastoma CSCs in the presence of sublethal doses of temozolomide or radiation, followed by analysis of epigenetic changes (ATAC-seq, ChIP-seq for histone modifications) and transcriptional programmes (RNA-seq) in surviving cells and their progeny. Key question: are epigenetic changes maintained after removal of the cytotoxic stimulus?
2. Single-cell chromatin analysis. Use of scATAC-seq and scRNA-seq to identify cells with “memory” in patient tumours after therapy compared to tumours without prior treatment. Identification of specific epigenetic landscapes associated with resistance.
3. Lineage tracing studies. Use of genetic barcoding systems or CRISPR-mediated tracing to demonstrate that cells surviving therapy give rise to recurrent tumours and maintain epigenetic marks.
4. Functional “memory erasure” experiments. Use of epigenetic modifier inhibitors (e.g., EZH2 inhibitors, HDAC inhibitors, PRDM9 inhibitors) to test whether CSCs can be sensitised to therapy by disrupting “memory.”

Without such experiments, the “tumour memory” concept remains a heuristic analogy. We present it as a hypothesis-generating tool rather than an established biological fact.

## Immune landscape of glioblastoma: from “cold” microenvironment to the invasive margin

### Heterogeneity of the GB immune landscape: from “cold” to “warm”

The immune landscape of glioblastoma is a critically important parameter determining not only the prognosis of the disease but also the potential efficacy of immunotherapeutic interventions. The principal difference between GB and other types of solid tumours lies in the nature of lymphocytic infiltration and the balance of effector and suppressor mechanisms. In melanoma, MSI-positive colorectal cancer and some forms of lung cancer, high density of tumour-infiltrating lymphocytes (TILs) correlates with favourable prognosis and good response to immunotherapy (59–63). In these cases, the microenvironment is characterised as “hot” with a predominance of CD8+ cytotoxic lymphocytes, CD4+ T helpers and, in some cases, CD20+ B cells. In contrast, prostate cancer and especially GB are characterised by extremely low lymphocytic infiltration and dominance of immunosuppressive mechanisms (64, 65). In these tumours, regulatory T cells (Treg) and M2-macrophages predominate, creating a “cold” microenvironment associated with an unfavourable prognosis.

However, the simplified view of GB as an absolute “immune desert” does not reflect the full complexity of the picture. According to the TCGA classification, four molecular subtypes of GB are distinguished — proneural, neural, classical and mesenchymal — each with a different degree of immune infiltration and, potentially, different sensitivity to immunotherapy (66). The mesenchymal subtype is characterised by the most pronounced immunosuppression: high infiltration of M2-polarised macrophages, myeloid-derived suppressor cells, increased production of TGFβ, IL-10 and checkpoint molecules (PD-L1, CTLA-4). It is for this subtype that the concept of a “cold” microenvironment with functional inactivation of TILs is most valid. The classical subtype, often associated with EGFR amplification, demonstrates an intermediate level of immune infiltration, where both suppressor and effector elements may be present, making immunotherapy potentially more effective. The **proneural subtype** is usually associated with a “warmer” microenvironment: higher CD8+ TIL content, lower TGFβ and BMDC levels (67–69). However, T-cell exhaustion mechanisms persist in all subtypes, albeit to a lesser extent. Thus, the GB immune landscape is not static or universally “cold”; it varies depending on the molecular subtype, spatial localisation within the tumour and disease stage. This creates both challenges and opportunities for the development of personalised immunotherapeutic strategies.

### Immunosuppressive microenvironment of central tumour zones

In the central zones of GB, where hypoxia and lactate acidosis are pronounced, a microenvironment is formed that actively suppresses anti-tumour immunity and protects the CSC population. A key role in this process is played by bone marrow-derived cells (BMDCs), which migrate into the tumour along a concentration gradient of the chemokine CXCL12 (released by damaged tissues) and hypoxia-inducible factor (HIF) (48). Myeloid cells constitute up to 70–80% of all migrating leukocytes, whereas lymphocytes are present in significantly smaller numbers — no more than 5% (7, 8). This ratio reflects a fundamental feature of GB: the tumour actively recruits innate immune cells, which are then reprogrammed into suppressor phenotypes, while the adaptive immune response is suppressed.

BMDCs infiltrating the tumour undergo forced reprogramming into immunosuppressive phenotypes under the influence of microenvironmental signals. Monocytes and neutrophils are transformed into M2-macrophages and N2-neutrophils, which produce arginase-1, indoleamine-2,3-dioxygenase and reactive oxygen species, suppressing T-cell effector functions and inducing their apoptosis (10, 70, 71). Myeloid suppressor cells, constituting the bulk of infiltrating leukocytes, inhibit T-cell proliferation and contribute to their functional inactivation. Hypoxia — the major factor in GB biology — itself transforms BMDCs into immunosuppressive phenotypes (72), and lactate production due to the Warburg effect further enhances immunosuppressive effects, contributing to the maintenance of “tumour memory” (73).

The central cytokine modulating the immune landscape and maintaining tumour memory is transforming growth factor β (TGFβ). Sources of TGFβ include both differentiated GB cells and cells of the “cold” microenvironment: M2-activated microglia and macrophages, M2-neutrophils, myeloid suppressor cells, regulatory T cells and tumour stromal cells (74). Normally, TGFβ acts through the canonical SMAD signalling pathway, inducing apoptosis of damaged cells and suppressing inflammation. However, in GB, mutations in SMAD pathway components lead to loss of cytoregulatory influences, while the immunosuppressive action of the cytokine is preserved and even enhanced (75). TGFβ activates non-canonical mechanisms — MAPK, RhoA/Rac and, importantly, the PI3K/AKT/mTOR signalling axis, leading to increased intracellular β-catenin levels and maintenance of CSC “stemness” (58).

Regulatory T cells (Treg, FoxP3^+^) play a special role in the central tumour zones, accumulating in the perivascular space, and their number directly correlates with tumour vessel density (76). Treg perform suppressor functions, limiting anti-tumour immunity through secretion of inhibitory cytokines (TGFβ, IL-10, IL-35) and metabolic competition for IL-2 (77). The presence of Tim-3^+^ Treg in the CSC microenvironment creates an “immune calm” zone, protecting the CSC pool from elimination. Significant numbers of regulatory B cells (Breg) are also found in the central zones, producing IL-10 and inhibiting the activity of dendritic cells and T killers (78). NK cells, which through activating receptors (NKG2D, NKp46) recognise markers of genome damage and induce CC apoptosis, also undergo suppression in the “cold” microenvironment (79). As a result, effector lymphocytes penetrating the central tumour zones are rapidly inactivated, and their anti-tumour potential is nullified.

It is important to note that in hypoxic niches, where TGFβ and lactate concentrations are maximal, effective lymphocytes are practically absent — they have either undergone apoptosis or been transformed into regulatory phenotypes (80, 81). Even if lymphocytes are preserved, they do not proliferate under such challenging conditions. The angiogenic (perivascular) niche differs from the hypoxic one by a higher oxygen content; however, cell proliferation here is directly linked to the formation of the vascular bed, and this niche serves as a critical sanctuary for CSCs (81). However, even here lymphocytes do not find favourable conditions for a full anti-tumour response — Treg and other suppressor cells effectively suppress their activity. This creates a fundamental obstacle to the implementation of anti-tumour immunity in the central tumour zones and explains why traditional immunotherapeutic approaches often prove ineffective in GB.

### The invasive margin as a putative sanctuary for functionally active lymphocytes

**Figure 2:**
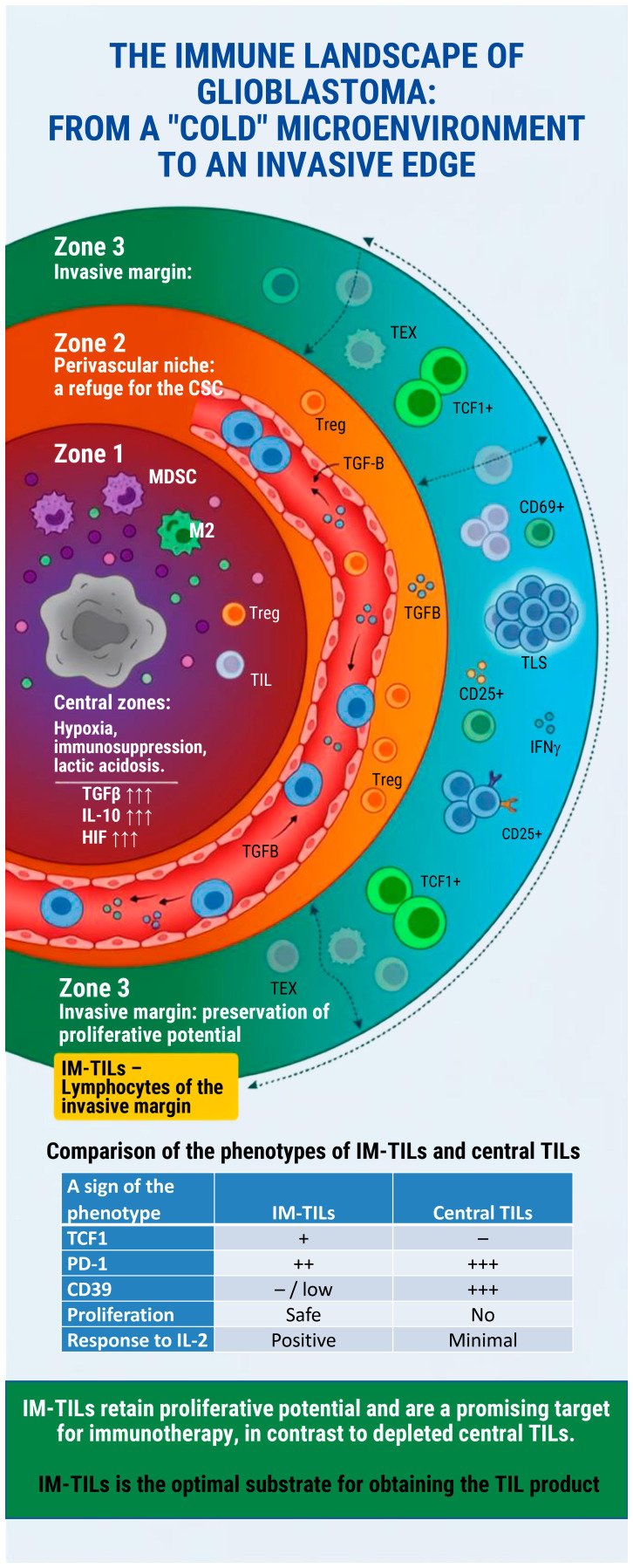
The immune landscape of glioblastoma: from a “cold” microenvironment to the invasive margin. The image provides a schematic representation of the spatial heterogeneity of the immune microenvironment in glioblastoma, divided into three functionally distinct zones. Zone 1 (central zones) is characterised by hypoxia, immunosuppression and lactate acidosis, with high concentrations of TGFβ, IL-10 and HIF. This “cold” microenvironment is dominated by myeloid-derived suppressor cells (MDSC), M2-polarised macrophages and regulatory T cells (Treg). Effective lymphocytes are virtually absent, and those present are terminally exhausted (TCF1^-^, PD-1^+++^, CD39^+++^). Zone 2 (perivascular niche) serves as a refuge for CSCs, which interact with endothelial cells and Treg. Zone 3 (invasive margin) represents the transition zone between the tumour and healthy brain tissue, where conditions approach normoxia. Here, functional lymphocytes are preserved, including TCF1^+^ (TPE) cells that retain proliferative potential. The invasive margin also contains tertiary lymphoid structures, activated lymphocytes (CD69^+^, CD25^+^) and IFNγ-producing cells. The table on the right compares the phenotypes of invasive margin TILs (IM-TILs) and central TILs. IM-TILs are characterised by TCF1^+^ expression, moderate PD-1 levels, low/negative CD39, preserved proliferative activity and positive response to IL-2 ex vivo, whereas central TILs are TCF1^-^, PD-1^+++^, CD39^+++^, non-proliferative and minimally responsive to IL-2. The lower panel emphasises that IM-TILs, which retain proliferative potential, represent the optimal substrate for obtaining a TIL-based biomedical cell product. TIL, tumour-infiltrating lymphocyte; IM-TIL, invasive margin TIL; MDSC, myeloid-derived suppressor cell; Treg, regulatory T cell; CSC, cancer stem cell; TGF-β, transforming growth factor beta; HIF, hypoxia-inducible factor; IL, interleukin; IFN-γ, interferon-gamma; TPE, T-cell progenitor exhausted; TEX, terminally exhausted T cell.

In contrast to the central tumour zones, the invasive margin — the transitional area between the neoplasm and healthy brain tissue — is a zone where a population of lymphocytes that may not have lost their anti-tumour potential could be preserved. In this region, the partial pressure of oxygen approaches that of normal brain tissue, and the concentrations of TGFβ and lactate are significantly lower than in the central hypoxic niches (**Figure 2**). It is here that the main confrontation between the immune system and the tumour unfolds, and it is here that lymphocytes still capable of an anti-tumour response might be detected.

Initially, lymphocytes infiltrating the invasive margin display signs of activation: expression of CD69, CD25, PD-1 and production of IFNγ. However, chronic antigenic stimulation combined with paracrine TGFβ action leads to their functional degradation. At the invasive margin, TGFβ transforms T cells into Treg, and chronic antigenic stimulation leads to functional degradation and exhaustion of lymphocytes under the influence of the transcription factor TOX (82, 83). TOX causes hyperexpression of inhibitory receptors — PD-1, LAG-3, TIM-3 and TIGIT — accompanied by reduced proliferative activity and suppressed synthesis of IL-2, IFNγ and TNFα (84). However, as shown in studies on T-cell exhaustion, the TIL population is not monolithic.

Based on the expression of the transcription factor TCF1, TILs can be divided into two subpopulations: TCF1^+^ (TPE) — long-lived, retaining the ability to proliferate and self-renew, and TCF1^-^ (TEX) — terminally exhausted cells with direct cytolytic activity but a limited lifespan (85, 86). Critically, it is at the invasive margin, under conditions approaching normoxia, that the subpopulation of TCF1^+^ TPE cells may be preserved, which may not have lost its proliferative potential. These cells, in contrast to central TILs with a TCF1^-^, PD-1^+^, LAG-3^+^, TIM-3^+^, CD39^+^ phenotype, which barely proliferate and are often apoptotic, could serve as a substrate for obtaining a biomedical cell product (87, 88).

The hierarchy of T-cell exhaustion at the invasive margin is also linked to the differentiation of memory cells. As shown above, T memory stem cells (TSCM), localised in specific niches and maintaining their own pool through self-renewal (89), represent the earliest and most long-lived subpopulation. At the invasive margin, where conditions approach normoxia, the preservation of cells with a phenotype close to TSCM may be possible, which fundamentally distinguishes this zone from the central hypoxic niches where terminally exhausted effectors predominate.

We hypothesise the existence of a therapeutically significant subpopulation and propose to designate it as “invasive margin lymphocytes” (IMLs). The proposed immunophenotype of IMLs, based on data from the TPE/TEX model and immunosuppression in GB, can be characterised as follows. Surface markers include CD3^+^, CD8^+^ (predominant), CD4^+^ (minority), PD-1^+^ (high), TCF1^+^ (TPE subpopulation), CD39^-^/low, CCR7^-^, CD69^+^/^-^ (activated but not resident memory) and CXCR3^+^. Transcription factors: TOX^+^ (high), TCF1^+^ (in a subset capable of proliferation), Eomes^+^/^-^, Blimp-1^+^ (exhaustion signature). Functional characteristics: reduced production of IL-2, IFNγ, TNFα; preserved cytotoxic activity ex vivo (after release from suppression); limited proliferative capacity but presence of a TCF1^+^ progenitor pool capable of expansion. In contrast to IMLs, central TILs are more exhausted and exhibit a TCF1^-^, PD-1^+^, LAG-3^+^, TIM-3^+^, CD39^+^ phenotype, barely proliferate and are often apoptotic. The difference from classical memory T cells (TSCM) is that IMLs do not express CCR7 (no homing to lymph nodes), and their program is dictated by chronic stimulation rather than physiological memory.

The following experimental criteria for IML identification have been proposed (90). Topographic criterion: the sample must be taken from the zone adjacent to healthy brain tissue. Immunophenotypic criterion: flow cytometry should reveal CD45^+^, CD3^+^, CD8^+^, PD-1^+^ (moderate/high), TCF1^+^ (fraction >10%), CD39^-^, CCR7^-^. Functional criterion: ability to reactivate and proliferate *in vitro* in the presence of IL-2 and anti-CD3.

It is important to emphasise that the traditional term “TILs” does not reflect spatial heterogeneity and does not single out the subpopulation that may retain therapeutic potential. IMLs, unlike the majority of TILs in GB, could serve as a substrate for producing a biomedical cell product, which has direct clinical implications. The introduction of a separate term is necessary for developing precision tissue-harvesting protocols and for subsequent comparison of results across studies. Admittedly, the proposed immunophenotype is based on extrapolation of data from models of T-cell exhaustion in other cancer types and the limited publications on TILs in GB. There is no direct evidence (single-cell sequencing of human IMLs); we regard this phenotype as a working hypothesis requiring validation in future studies (see Section 6.4).

### Knowledge gaps and validation requirements for the IML concept

The proposed concept of “invasive margin lymphocytes” (IMLs) is a working hypothesis based on extrapolation of data from models of T-cell exhaustion in other cancer types and the limited publications on TILs in glioblastoma. Direct evidence for the existence of this subpopulation in human GB patients is currently lacking. To confirm or refute the IML concept, the following studies are necessary:

1. Spatial transcriptomics. Use of spatial sequencing methods (e.g., 10x Visium, Slide-seq) to map transcriptional profiles of lymphocytes in different tumour zones (centre, perivascular niche, invasive margin). This would allow identification of cells with a TCF1^+^ phenotype in the invasive zone.
2. Multiplex immunofluorescence with topographic localisation. Use of multiplex immunohistochemistry (e.g., CODEX, MIBI) for simultaneous detection of CD3, CD8, PD-1, TCF1, CD39, CCR7 in tissue sections with preserved spatial information. This would allow visualisation of the putative IML subpopulation in the invasive margin and comparison with central TILs.
3. Single-cell sequencing from different tumour zones. Isolation of lymphocytes from surgical samples taken from the invasive margin and central zones, followed by transcriptomic (scRNA-seq) and chromatin accessibility (scATAC-seq) analysis. Identification of cells with the transcriptional signature TCF1^+^ PD-1^+^ CD39^-^ CCR7^-^.
4. Functional ex vivo studies. Isolation of lymphocytes from the invasive margin and testing their ability to proliferate in the presence of IL-2 and anti-CD3. Comparison of proliferative potential with central TILs.
5. Correlation with clinical outcomes. Analysis of the association between the presence of IMLs (by immunohistochemistry) and overall and relapse-free survival in GB patients.

Without such studies, the IML concept remains hypothetical. We present it as a heuristic tool for planning future experiments and developing therapeutic strategies, rather than as an established biological fact.

Thus, the invasive margin of GB represents a dynamic demarcation line between neoplasm and healthy tissue, where a therapeutically significant subpopulation of lymphocytes may be preserved. It is IMLs, and primarily the putative TCF1^+^ (TPE) subpopulation, that could constitute the real substrate for obtaining a TIL product, as they may have retained proliferative potential and could be expanded ex vivo. This makes the invasive margin not just a zone of interest for fundamental immunology but a key potential source of material for personalised immunotherapy of GB.

## TSCM — a key tool for suppressing tumour memory

### Hierarchy of T-cell memory and the therapeutic value of TSCM

Lymphocytes, as central effectors of adaptive immunity, perform not only standard functions of eliminating pathogens and tumour cells but also play a critical role in regulating the activity of stem cells in tissue niches. At the organismal level, they act as sensors of imperfect repair processes, eliminating cells with critical genomic damage. In response to DNA damage, tumour cells activate stress signalling and produce chemokines CXCL9, CXCL10, CXCL12, along the gradient of which lymphocytes infiltrate the tumour. This process is one of the key components of anti-tumour surveillance; however, in GB it proves insufficiently effective due to the powerful immunosuppression created by the microenvironment.

The TIL population consists mainly of T cells, among which cytotoxic T lymphocytes (CD8+), T helpers (CD4+) and regulatory T cells (Treg) perform various but complementary functions. Up to 95% of TILs express the αβ T-cell receptor (TCR), enabling them to recognise antigens presented on major histocompatibility complex (MHC) class I molecules (CD8+ T cells) or class II molecules (CD4+ T cells) (91). After primary contact with a tumour antigen, some T cells form a memory cell pool that provides long-term immunological protection (92).

The progenitor of all T-cell memory subpopulations is the naïve T cell. After encountering a specific antigen, it forms a heterogeneous population of memory cells, in which four main subpopulations are distinguished: T memory stem cells (TSCM), central memory T cells (TCM), effector memory T cells (TEM) and terminally differentiated effector memory T cells expressing CD45RA (TEMRA). At the base of the memory cell hierarchy lie TSCM (CD3+ TCR+), which are localised in specific niches (lymph nodes, bone marrow) and maintain their own pool through self-renewal (89). This is achieved by activation of the canonical WNT/β-catenin signalling cascade, the PI3K/AKT/mTOR pathway and other mechanisms that ensure the preservation of “stemness” and prevent differentiation. TSCM are characterised by the phenotype CD45RA+ CD45RO− CCR7+ CD62L+ CD95+ CD27+ CD28+ and possess a unique ability for self-renewal and multipotency, making them the most long-lived and therapeutically valuable subpopulation.

TCM (CD45RO+ CD45RA− CD62L+ CD95+ CD27+ CD28+) lose some “stem” properties but retain high proliferative potential and a long lifespan (93). They are localised in lymph nodes and upon re-encounter with antigen are able to rapidly proliferate and differentiate into effector cells. TEM (CD45RO+ CD45RA− CCR7+ CD62L+ CD95+ CD27+ CD28+) and TEMRA (CD45RA+ CD62L− CD27− CD28− CD95+) are effector T lymphocytes (94, 95). They constitute the majority of TILs in the tumour, but their distinguishing feature is rapid exhaustion and death. The relationship between naïve and mature memory T cells follows the principle of ontogenetic continuity, in which excessive accumulation of mature effector cells limits TSCM proliferation, preventing their exhaustion and ensuring long-term maintenance of the memory cell pool.

The hierarchical organisation of T-cell memory has direct therapeutic significance. TSCM, possessing the highest proliferative potential and ability for long-term persistence, represent the most attractive subpopulation for adoptive immunotherapy. It is TSCM that provide long-term anti-tumour immunity and are capable of repopulating the effector pool upon re-encounter with antigen. Therefore, strategies aimed at preserving or increasing the proportion of TSCM in the TIL product are critically important for the success of immunotherapy.

### Molecular mechanisms of immunological memory formation

The formation of the memory cell pool implies that during primary contact with antigen, the naïve T cell undergoes metabolic stress, which triggers a cascade of epigenetic and transcriptional rearrangements. Induced DNA damage is recognised by repair enzymes that function as epigenetic regulators, facilitating the fixation of “epigenetic scars” during the primary immune response (96). During repair, genomic loci that were functionally active during the response are marked and converted to an open chromatin state (97). Subsequently, this “record” becomes a marker of accelerated activation upon re-exposure to antigen. Thus, immunological memory should be defined as a dynamically maintained chromatin state: a continuum of stable genetic recombination and a plastic epigenetic landscape (98). A high capacity of DNA repair systems is the main condition for TSCM longevity, while a decrease in their efficiency leads to the differentiation of TSCM into short-lived effectors (99).

Regulatory functions of CD8+ lymphocytes are executed in close collaboration with CD4+ helpers, which “license” dendritic cells — i.e., convert them from an immature to an active state (100). Only a licensed dendritic cell can provide a naïve T cell with all three necessary signals: antigen, co-stimulation and IL-12, thereby ensuring priming and preventing exhaustion (101). The process is initiated by interaction of CD40L on the CD4+ helper with the CD40 receptor on the dendritic cell, triggering the production of pro-inflammatory cytokines, induction of co-stimulatory molecules and creation of a chemokine gradient to attract T lymphocytes to the contact zone (102). Consequently, both lymphocyte types — CD4+ and CD8+ — recognise their specific antigens on the same dendritic cell, completing the formation of effector and memory cell pools (103).

Besides their helper functions, CD4+ cells also include a subpopulation with cytolytic activity (CD4+ CTL). These cells express the regulatory factors Eomes and Runx3, as well as markers NKG2D, NKG7 and CRTAM (104). Their cytotoxic activity targets antigen-presenting cells and tumour cells. Under the influence of IFNγ from CD8+ cytotoxic T cells and type 1 CD4+ T helpers, these cells start to aberrantly express MHC-II. This expands the repertoire of anti-tumour immunity and creates additional opportunities for eliminating tumour cells that evade the CD8+ T-cell response. In light of this, the boundaries between “helper” and “effector” functions of TILs depend on the microenvironmental context and the state of the tissue niche (105). In particular, T follicular helper cells (Tfh) have been described that coordinate the formation of tertiary lymphoid structures within the tumour by secreting the chemokine CXCL13, thereby creating additional homing vectors for T cells (106, 107).

### Role of γc-family cytokines in maintaining TSCM and suppressing tumour memory

The functioning of TSCM and their therapeutic potential are largely determined by the cytokine microenvironment. A key role in maintaining the TSCM pool is played by γc-family cytokines (common gamma chain): IL-2, IL-7, IL-15 and IL-21. Each of these cytokines exerts a specific effect on TSCM, which has direct implications for the development of immunotherapy strategies aimed at suppressing tumour memory.

IL-2 is a classical cytokine that promotes proliferation and terminal differentiation of TSCM (89). However, its action is dual: on the one hand, IL-2 is necessary for TIL expansion; on the other, excessive IL-2 stimulation leads to depletion of the TSCM pool and accumulation of effector cells with a limited lifespan. Therefore, in TIL therapy protocols, IL-2 dosage must be carefully balanced, especially for GB, where TILs are characterised by a high degree of exhaustion. IL-7 and IL-15 play critically important roles in the homeostasis and survival of TSCM (89). IL-7 is essential for the survival of naïve T cells and memory cells, whereas IL-15 supports homeostatic proliferation of TSCM and prevents their apoptosis. Both cytokines contribute to the maintenance of the “stem” phenotype of TSCM, making them important components of TIL culture media. IL-21 is a key cytokine for TSCM generation and acts as a synergist in maintaining T-cell “stemness” (89). IL-21 enhances TSCM expansion in combination with IL-15 and promotes the formation of long-lived memory cells. Furthermore, IL-21 enhances the cytotoxic activity of NK cells, which may contribute to the elimination of CSCs (79).

The hypothetical effect of γc-cytokines on CSCs is of particular interest (**Table 2**). IL-2 may induce CSC differentiation, which potentially reduces their tumourigenic potential but may simultaneously promote heterogenisation of the tumour population. IL-7 and IL-15, as shown in models of other tumours, promote “warming up” of the microenvironment, enhancing T-cell recruitment and activation, which may disrupt the CSC niche. IL-21, by enhancing NK cell activity, may promote direct elimination of CSCs. However, it should be emphasised that these effects are hypothetical and require experimental validation.

**Table 2:** Effect of γc-cytokines on TSCM and potential impact on CSCs.

Cytokine	Effect on TSCM	Hypothetical/known effect on CSCs
IL−2	Promotes proliferation and terminal differentiation of TSCM. Leads to exhaustion of the TSCM pool and accumulation of effector cells	CSC differentiation
IL−7	Preserves the TSCM pool. Critically important for survival of naïve and memory T cells	Microenvironment warming, recruitment and activation of T cells
IL−15	Homeostasis and expansion of TSCM	(same as IL−7)
IL−21	Key cytokine for TSCM generation. Synergist for maintaining T-cell “stemness”	Elimination of CSCs via NK cells

Thus, γc-cytokines represent a powerful tool for modulating the TSCM pool and, potentially, for suppressing tumour memory. The combination of IL-7, IL-15 and IL-21 in TIL expansion protocols can significantly increase the yield of cells with a TSCM phenotype, thereby enhancing the efficacy of adoptive immunotherapy. Furthermore, modulation of the cytokine microenvironment using these cytokines may directly affect CSCs, disrupting their niche and reducing their ability to preserve and transmit tumour memory.

### From TSCM to TIL therapy: therapeutic perspectives

The link between TSCM and CSC tumour memory opens fundamentally new therapeutic perspectives. If TSCM are carriers of long-term immunological memory, ensuring an effective response upon re-encounter with antigen, then CSCs act as carriers of tumour memory, ensuring survival and recurrence. Remarkably, both cell types employ convergent mechanisms — WNT/β-catenin signalling, DNA repair and epigenetic regulation — allowing them to be viewed as evolutionarily related memory systems. From a therapeutic perspective, drugs that disrupt WNT signalling or DNA repair in TSCM (e.g., PARP inhibitors, WNT inhibitors) may exert similar effects on CSCs, opening possibilities for targeting both cell types simultaneously.

However, a more promising approach is the use of adaptive immunity — and in particular TIL therapy — for the targeted elimination of CSCs. TSCM, due to their ability for self-renewal and the formation of long-term anti-tumour immunity, can serve as a “living drug” that not only eliminates existing CSCs but also prevents the formation of new carriers of tumour memory. In this context, TIL therapy oriented towards preserving and enhancing the TSCM population can be viewed as a strategy for managing tumour memory, rather than merely as a method of enhancing the cytotoxic response.

A key practical conclusion is the need to adapt existing TIL therapy protocols for GB, taking into account the described mechanisms. The use of a combination of γc-cytokines (IL-7, IL-15, IL-21) during TIL expansion, the use of GSK-3β inhibitors to activate WNT signalling and preserve TSCM, and blockade of TGFβ to prevent exhaustion — all these approaches can significantly increase the therapeutic potential of the TIL product. Furthermore, selection of TILs by TSCM phenotype (CD45RA+ CD45RO− CCR7+ CD62L+ CD95+ CD27+ CD28+) can ensure the production of a cell product with optimal properties for long-term persistence and anti-tumour activity.

It is important to emphasise that a direct causal relationship between TSCM and CSC programs has not yet been proven, and the proposed analogy serves rather as a heuristic tool for identifying new targets than as a mechanistic hypothesis. However, it is precisely this analogy that allows us to rethink TIL therapy and consider it not just as a method of cytoreduction but as a strategy for managing cellular memory — both immunological and tumour.

## Active immunotherapy: cancer vaccines and “warming up” the microenvironment

### Pharmacological modulation of the microenvironment: overcoming immunosuppression

As shown in the previous sections, TGFβ is a key mediator of immunosuppression in the GB microenvironment, maintaining tumour memory through activation of the WNT/β-catenin signalling pathway in CSCs, induction of Treg and exhaustion of effector T cells. This makes TGFβ a highly attractive but at the same time complex therapeutic target. The idea of direct pharmacological inhibition of TGFβ using galunisertib, vactosertib or trabederisen has, unfortunately, not lived up to expectations (108). The tumour rapidly activates alternative survival pathways, negating the effect of monotherapy. Indirect targeting of TGFβ signalling through inhibition of HIF (hypoxia-inducible factor) appears more promising; however, belzutifan and other HIF inhibitors have not shown convincing survival benefits in clinical trials (109).

Certain prospects are associated with antagonists of the CXCR4/CXCL12 axis, in particular plerixafor (AMD3100). This drug reduces the number of BMDCs in the tumour and, in combination with checkpoint inhibitors, may enhance lymphocytic infiltration (110). Importantly, the reduction of the myeloid suppressor cell pool creates favourable conditions for TIL activation and can be considered an important stage of preparation for adoptive immunotherapy. Furthermore, a number of epigenetic modulators, including the PARP inhibitor olaparib, the EZH2 inhibitor tazemetostat, the demethylating agent decitabine and the histone deacetylase inhibitor valproic acid, are capable of enhancing lymphocytic infiltration of the tumour, inducing genomic instability and reducing CSC plasticity (111–114). The effect of these drugs on the hierarchy of tumour cells and “tumour memory” is practically unstudied, but their integration with immunotherapy methods opens significant therapeutic prospects.

Thus, pharmacological support should be aimed not so much at the direct destruction of CSCs as at creating an immunogenic microenvironment in which TILs can function effectively. This includes suppression of suppressor cells (Treg, MDSC, M2-macrophages), blockade of inhibitory molecules (PD-1, CTLA-4) and enhancement of chemotactic signals to attract effector lymphocytes. This approach, known as “warming up” the TME, is a necessary condition for the success of subsequent adoptive TIL therapy.

### Cancer vaccines as a tool for inducing anti-tumour immunity

Cancer vaccines represent a powerful tool of active immunotherapy, capable of inducing a T-cell response and forming a new memory cell pool that competes with CSC tumour memory. Theoretically, vaccination should not only stimulate the elimination of existing tumour cells but also create long-term immunological surveillance, preventing recurrence. In clinical practice for GB, three main types of cancer vaccines are used: dendritic cell, peptide and mRNA vaccines. Each of these approaches has its own advantages and limitations, but all are aimed at one strategic task — the transformation of the “cold” GB microenvironment into a “warm” one capable of supporting an effective anti-tumour immune response.

Dendritic cell vaccines use autologous dendritic cells loaded with tumour antigens to prime naïve T cells. The most studied vaccine is DCVax, which in a phase III clinical trial showed an increase in overall survival in GB of 3 months, with the proportion of five-year survivors being 13.0% versus 5.7% in the control group (115). The ICT-107 vaccine, targeted at antigens expressed by CSCs, increased overall survival by 2.2 months but proved effective only in HLA-A1/A2 carriers (116, 117). The vaccine based on Wilms’ tumour protein (WT1) mRNA demonstrated the ability to induce a T-cell response associated with clinical improvement (118). The success of dendritic cell vaccines is related both to direct antigen presentation and to the formation of a new memory cell pool that can potentially compete with CSCs for niches in the microenvironment.

Peptide vaccines introduce ready-made epitopes of neoantigens into the body, simplifying the presentation process and enhancing T-cell infiltration of the tumour. However, clinical experience with peptide vaccines in GB has been mixed. Rindopepimut, the best-known vaccine against the EGFRvIII mutation, did not show a survival benefit, as the tumour simply “shed” the EGFRvIII antigen and continued to grow (119). A similar result was obtained with peptide vaccines against IDH1 (R132H) and H3.3 K27M, indicating high CSC plasticity and their ability to evade immune responses by losing or mutating target antigens. Nevertheless, the IDH1-vac vaccine provided three-year control in more than 80% of patients in limited studies (120, 121). The main drawback of peptide vaccines is the need for potent adjuvants (e.g., GM-CSF) and narrow specificity limited to certain HLA alleles.

mRNA vaccines appear to be the most promising direction due to their high personalisation potential and rapid production capability. Theoretically, an mRNA vaccine can be created for each patient individually based on sequencing of their tumour genome, followed by identification of unique neoantigens. However, in practice, classical antigens are also used; for example, the CVGBM vaccine, encoding 8 epitopes from 4 antigens (p53, Survivin, TLR4, TRP2), is at the preclinical development stage (122). Prototypes of mRNA vaccines directed against a number of hypothetical targets obtained by bioinformatics methods are being actively developed (72, 123, 124). The use of mRNA vaccines enhances lymphocytic infiltration of the tumour and creates favourable conditions for subsequent adoptive TIL therapy (125).

### Viral vectors for antigen delivery and TME “warming up”

The use of viruses to deliver antigens to tumour cells represents one of the most elegant and biologically sound approaches. Adenoviruses, canarypox viruses or herpes viruses, into whose genomes DNA sequences enhancing tumour immunogenicity can be inserted, penetrate GB cells and force them to synthesise and “display” on their membrane the very antigens against which the immune response is subsequently directed. This approach, in effect, turns tumour cells into “factories” for antigen production, significantly enhancing their immunogenicity and facilitating recognition by TILs.

Theoretically, autologous mononuclear cells capable of migration and homing to the neoplastic focus could serve as vehicles for virus delivery (126). This allows the blood-brain barrier to be overcome and the virus to be delivered directly to the tumour microenvironment. However, in response to viral attack, the tumour begins to defend itself by expressing immune checkpoint proteins (PD-L1), which requires pharmacological support with checkpoint inhibitors. It is important to note that the combination of “virus + cancer vaccine” may be insufficient for complete “warming up” of the microenvironment; active immunotherapy technologies must be complemented by adoptive immunotherapy methods that ensure the final elimination of CSCs (127, 128).

### Three-stage strategy for CSC regulation and its rationale

Thus, active immunotherapy using cancer vaccines and viral vectors performs the critically important function of “warming up” the microenvironment — creating an immunogenic landscape in which TILs can function effectively. However, none of the described methods is self-sufficient; cancer vaccines can induce a T-cell response and enhance tumour infiltration, but they do not ensure the direct elimination of CSCs in sufficient quantity. Similarly, pharmacological modulation of the TME can reduce immunosuppression but does not create long-term cellular immunity.

The culmination of the proposed strategy is the adoptive transfer of TILs obtained from the invasive margin. This approach allows the advantages of active immunotherapy (induction of antigen-specific response, “warming up” of the TME) to be combined with the power of adoptive therapy (transfer of a large number of functionally active cells capable of long-term persistence). In this context, active immunotherapy and pharmacological support serve as necessary preparatory stages, creating optimal conditions for engraftment and functioning of adoptively transferred TILs.

**Figure 3:**
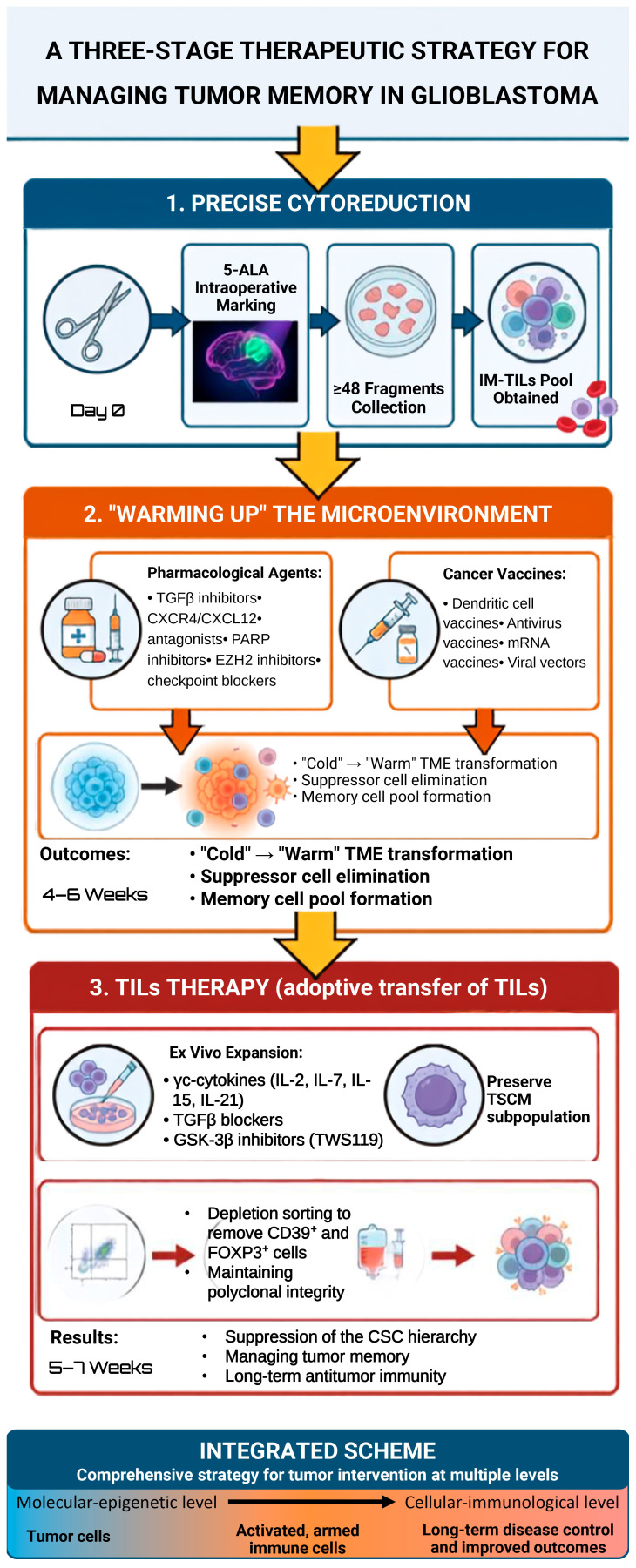
A three-stage therapeutic strategy for managing tumour memory in glioblastoma. The image presents the proposed three-stage therapeutic algorithm integrating precision surgery, active immunotherapy and adoptive TIL therapy. Stage 1 (Precision cytoreduction) involves intraoperative marking using 5-aminolevulinic acid (5-ALA), collection of ≥48 tumour fragments from the invasive margin, and obtaining the IM-TIL pool as the source material. Stage 2 (“Warming up” the microenvironment) comprises pharmacological modulation (TGFβ inhibitors, CXCR4/CXCL12 antagonists, PARP inhibitors, EZH2 inhibitors, checkpoint blockers), cancer vaccines (dendritic cell vaccines, peptide vaccines, mRNA vaccines) and viral vectors. The outcomes include transformation of the “cold” TME into a “warm” one, elimination of suppressor cells and formation of a memory cell pool. Stage 3 (Adoptive TIL therapy) involves ex vivo expansion of TILs using γc-cytokines (IL-2, IL-7, IL-15, IL-21), TGFβ blockers and GSK-3β inhibitors (TWS119) to preserve the TSCM subpopulation, followed by depletion sorting to remove CD39^+^ and FOXP3^+^ cells while maintaining polyclonality. The final outcomes include suppression of the CSC hierarchy, management of tumour memory and establishment of long-term anti-tumour immunity. The lower panel integrates the three stages into a comprehensive strategy for tumour intervention at multiple levels: from the molecular-epigenetic level to the cellular-immunological level, ultimately achieving long-term disease control and improved outcomes. 5-ALA, 5-aminolevulinic acid; IM-TIL, invasive margin TIL; TME, tumour microenvironment; TIL, tumour-infiltrating lymphocyte; TSCM, T memory stem cell; TGF-β, transforming growth factor beta; IL, interleukin; GSK-3β, glycogen synthase kinase 3 beta; CSC, cancer stem cell.

The proposed three-stage strategy includes: 1) precision cytoreduction of the invasive margin with material harvesting for TIL production; 2) “warming up” the microenvironment using cancer vaccines, viral vectors and pharmacological agents; 3) adoptive transfer of TILs derived from the IML pool to suppress the CSC hierarchy and manage tumour memory. Such a comprehensive strategy allows attack on the tumour at multiple levels: from molecular-epigenetic (pharmacological modulation) to cellular-immunological (vaccination and TIL therapy), creating maximum chances for durable disease control (**Figure 3**).

## TIL therapy: advantages, drawbacks and adaptation of protocols for glioblastoma

### Technological platform of TIL therapy: from melanoma to glioblastoma

Therapy using tumour-infiltrating lymphocytes represents one of the most personalised and biologically sound methods of adoptive immunotherapy. Most technological steps — isolation, culture, selection and functional testing of TILs — have been developed and validated in the treatment of melanoma, where TIL therapy has demonstrated clinical efficacy, including phase III results (59–61). However, for GB, the number of successful clinical studies of TILs remains extremely limited, which is due to the unique features of the tumour microenvironment: a high degree of T-cell exhaustion, dominance of immunosuppressive mechanisms and low baseline TIL density in tumour tissue (129). Direct transfer of protocols from melanoma to GB is not trivial and requires substantial adaptation.

The process of producing a biomedical cell product based on TILs is a multi-step biotechnological procedure. Key steps include isolation of TILs from tumour tissue, primary expansion (pre-REP), selection of target populations, rapid expansion (REP) and subsequent transplantation of the obtained cells into the patient (130). The technological platform is based on protocols standardised for melanoma; however, for GB, fundamental revision of each stage is required, taking into account the higher proportion of exhausted cells and the pronounced suppressive action of TGFβ.

Primary TIL expansion (pre-REP) is initiated at the surgical intervention stage. The functional architecture of the CSC microenvironment is spatially heterogeneous, and the selection of the tumour site for material harvesting is critically important. In central hypoxic niches, where TGFβ and lactate concentrations are maximal, effective lymphocytes are virtually absent — they have either undergone apoptosis or been transformed into Treg. The main source of therapeutically significant TILs is the invasive margin of the tumour, where the putative IML subpopulation that may not have lost its proliferative potential is preserved. Therefore, protocols for GB must include topographic marking of material harvesting to ensure that samples are obtained specifically from the invasive zone.

Culture of tumour fragments is carried out in the presence of IL-2 (6000 IU/mL) under standard conditions (37 °C, 5% CO_2_). However, for GB, due to the low baseline number of TILs and their high degree of exhaustion, an increase in the number of fragments (more than 48) and the mandatory addition of TGFβ blockers (galunisertib, fresolimumab) and γc-cytokines (IL-7, IL-15, IL-21) are required (129, 131). The use of a cocktail of IL-2, IL-15 and IL-21 in combination with OKT3 allows effective production of TILs from GB with low levels of exhaustion markers (131). Culture duration should be minimal, as early exhaustion of the TCF1^+^ precursor pool is a major problem. Accordingly, the use of **“young” TILs** obtained after a short (10–14 days) pre-REP stage has been proposed, which allows avoidance of terminal differentiation and yields cells with higher proliferative potential (132).

### Selection and functional testing of TILs

A critically important component of TIL product manufacturing is precision selection of the target population. In the case of GB, phenotype selection becomes especially important, as Treg and exhausted clones dominate the TIL population. Preferred for further work are TILs expressing markers associated with an effective anti-tumour response. The antigen CD137 has shown the greatest efficacy for GB, being expressed at high levels on activated but not exhausted T cells (133).

The central task of selection is the isolation of the TSCM population of phenotype CD45RA^+^ CD45RO^-^ CCR7^+^ CD62L^+^ CD95^+^ CD27^+^ CD28^+^, which possesses the highest proliferative potential and ability for long-term persistence. However, overly stringent sorting carries the risk of losing the key advantage of TILs — polyclonality, which is necessary to overcome tumour heterogeneity and prevent immune “escape.” In this regard, it is advisable to perform not positive but depletion sorting, removing terminally exhausted cells (CD39^+^) and regulatory populations (FOXP3^+^), which reduces the risk of losing polyclonality and preserves cell population heterogeneity.

Furthermore, it is advisable to include incubation of cells with GSK-3β inhibitors (e.g., TWS119, CHIR99021), which mimic activation of the WNT signalling pathway — a key mechanism for preserving “stemness” (134). Application of such inhibitors at the TIL expansion stage can significantly increase the yield of cells with a TSCM phenotype, enhancing the therapeutic potential of the final product.

In addition to phenotypic characteristics, the molecular-genetic profile of TILs is of great importance. A number of genes have been described whose expression is associated with reduced therapeutic potential: TOB and KLF2 suppress IL-2 production; Ski/Sno family genes maintain T-cell quiescence; ERF inhibits lymphocyte migration into the tumour; REST suppresses PI3K signalling (135). The absence of the transcription factor TCF-1 deprives cells of long-term persistence, whereas factors Bach-2, FOXO1 and STAT3 can directly block cytotoxic activity. However, accounting for the full spectrum of these factors during routine product preparation seems incredibly labour-intensive, even with the use of artificial intelligence and automated cell rejection methods based on undesirable transcriptional signatures (136). Technological complexity negates the advantages of TILs, turning the method into a more expensive and less effective version of CAR-T therapy.

Functional testing is mandatory. TIL samples from each well are cultured with autologous tumour cells, as well as with HLA-matched (positive control) and HLA-mismatched (negative control) cell lines. The level of IFNγ production in the supernatant after 24 hours is determined by ELISA. TILs that react with autologous or HLA-matched cells are considered tumour-antigen specific; cells that react with HLA-mismatched lines are excluded from further processing (137).

Rapid expansion (REP) allows the production of a clinically significant quantity of TILs. Selected specific TILs are pooled and cultured with IL-2 (3000 IU/mL), agonistic anti-CD3 antibodies (30 ng/mL) and irradiated allogeneic peripheral blood mononuclear cells (feeder cells) at a 1:200 ratio (138). Feeder cells serve as a source of growth factors, ensuring more than 1000-fold increase in TIL numbers over 14 days. However, for GB, this stage requires optimisation: IL-2 concentrations, culture duration and feeder cell composition must be adapted to preserve the TCF1^+^ precursor pool and prevent terminal exhaustion.

### Types of TIL products: “young”, selected and unselected TILs

The final TIL therapy product can be represented by three main types, each with its own advantages and limitations in the context of GB.

“Young” TILs, obtained after minimal expansion (10–14 days, without selection and long culture), retain key properties: high levels of CD27 and CD28, long telomeres and a “naïve-like” phenotype CD45RA^+^CCR7^+^CD62L^+^CD27^+^CD28^+^ (132). These markers are characteristic of both naïve T cells and TSCM, making “young” TILs the closest to the therapeutically valuable stem subpopulation. “Young” TILs expanded in the presence of TGFβ blockers and a cocktail of IL-7/IL-15/IL-21 represent the greatest interest for GB therapy. They avoid terminal differentiation, retain polyclonality and are capable of effective expansion *in vivo* after adoptive transfer. In fact, this is a biomedical cell preparation of immune system stem cells for personalised regulation of GB CSC “memory.”

Unselected TILs recognise a wide range of antigens, including unknown neoantigens, but contain a large fraction of cells without anti-tumour activity. Long expansion, necessary to obtain a clinically significant quantity of such cells, leads to exhaustion, loss of TCF1^+^ precursors and accumulation of PD-1^+^ TIM-3^+^ TEX. Selected TILs target a limited set of antigens, bringing their efficacy closer to CAR-T cells, but their production is significantly more complex and expensive (137). Furthermore, selection may “age” cells, reducing their proliferative potential and ability for long-term persistence.

For GB, given the high heterogeneity of the tumour and the risk of loss of antigenic targets, “young” TILs or unselected TILs with a short culture period are preferred. Selected TILs may be used in cases where dominant neoantigens are known (138); however, their use requires a careful balance between specificity and the risk of immune escape.

### Clinical challenges of TIL therapy in GB

Preparation for TIL infusion is not limited to biotechnological steps. Existing protocols include a course of lymphodepleting therapy (cyclophosphamide and fludarabine for 2–7 days) to eliminate endogenous lymphocytes, including Treg competing for growth factors (139). The main goal of lymphodepletion is to create a systemic “immunological space” for optimal proliferation and functioning of adoptively transferred TILs. However, for GB, systemic lymphodepletion encounters a fundamental obstacle related to the blood-brain barrier (BBB). Although the BBB is disrupted in tumour tissue, its permeability remains heterogeneous. Systemic administration of cyclophosphamide and fludarabine, while effectively eliminating immune cells in the periphery, poorly penetrates the BBB (140). As a result, despite systemic immunosuppression, resistant Treg may persist in the tumour microenvironment and at the invasive margin, capable of neutralising the effect of infused TILs. This makes traditional lymphodepletion poorly effective for achieving a local anti-tumour response while exposing the patient to potentially unjustified systemic toxicity.

A possible solution to overcome the BBB and achieve local immunomodulation could be the use of an Ommaya reservoir for intraventricular administration of lymphodepleting agents and TILs; however, this strategy requires further experimental and clinical studies (34–36). Furthermore, in general oncology, high doses of IL-2 are administered after TIL infusion to support their proliferation (141). However, high doses of IL-2 can induce capillary leak syndrome requiring intensive care, sharply limiting the applicability of the method for frail and elderly patients. A potential alternative could be transduction of TILs with genes encoding IL-2 (142) synthesis; however, genetic modification negates the key advantages of TILs, bringing their safety profile closer to that of CAR-T cells.

The total time from tumour resection to obtaining the final product is 5–7 weeks, which is a critical limitation for GB patients, whose prognosis upon recurrence is measured in weeks. This circumstance dictates the need for the development of accelerated protocols for biomedical cell product manufacturing, including shortening of the REP stage, use of “young” TILs and optimisation of culture conditions for TSCM preservation.

### Protocol adaptation for GB: from challenge to solution

Given the described challenges, we propose the following key adaptations of TIL therapy protocols for GB:

First, material harvesting must be topographically oriented to the invasive margin of the tumour, where the putative IML subpopulation with a TCF1^+^ TPE phenotype may be preserved. This requires intraoperative marking of harvesting zones and the use of imaging methods to identify the transitional zone between the tumour and healthy tissue.

Second, the primary expansion stage should include TGFβ blockers (galunisertib, fresolimumab) and a cocktail of γc-cytokines (IL-7, IL-15, IL-21) to prevent exhaustion of TCF1^+^ precursors and preserve TSCM. Addition of GSK-3β inhibitors (TWS119, CHIR99021) may further enhance WNT signalling and increase the yield of cells with a TSCM phenotype.

Third, selection should be depletion-based rather than positive, to preserve polyclonality. The removal of CD39^+^ terminally exhausted cells and FOXP3^+^ Treg is preferable to positive selection of TSCM, which may lead to loss of heterogeneity.

Fourth, culture duration should be minimal (10–14 days for pre-REP and 10–14 days for REP), with an emphasis on obtaining “young” TILs that have retained proliferative potential. The use of autologous feeder cells instead of allogeneic ones may reduce the risk of alloreactivity and improve engraftment.

Fifth, lymphodepletion should be local (143) (via Ommaya reservoir) or replaced by targeted elimination of Treg using antibodies (e.g., anti-CD25), to minimise systemic toxicity and overcome the BBB.

### Clinical limitations and unresolved questions

The proposed three-stage strategy, while conceptually attractive, faces a number of practical challenges that require acknowledgement and further investigation.

1. TIL production time. The total time from tumour resection to obtaining the final cell product is 5–7 weeks under standard protocols. For patients with recurrent GB, whose prognosis is often measured in weeks, this is a critical limitation. The manuscript mentions “accelerated protocols,” but specific approaches require more detailed discussion. Possible solutions include: (a) use of “young” TILs with shortened culture duration (10–14 days instead of 5–7 weeks) (144); (b) use of automated culture systems (e.g., CliniMACS Prodigy) to accelerate the process; (c) cryopreservation of TILs at the pre-REP stage with subsequent expansion at recurrence. However, all these approaches require clinical validation and are not standardised.
2. Lymphodepletion and the BBB. Systemic lymphodepletion with cyclophosphamide and fludarabine, while effectively eliminating immune cells in the periphery, poorly penetrates the BBB. This creates the risk of resistant Treg persisting in the tumour microenvironment. Possible solutions: (a) local administration of lymphodepleting agents via an Ommaya reservoir; (b) use of targeted antibodies against Treg (e.g., anti-CD25) instead of systemic chemotherapy; (c) complete abandonment of lymphodepletion in favour of TME “warming” using cancer vaccines and pharmacological agents. However, all these approaches are experimental and lack an evidence base.
3. Toxicity of high-dose IL-2. High doses of IL-2 (600,000–720,000 IU/kg every 8 hours) are associated with capillary leak syndrome requiring intensive care. For elderly GB patients, who constitute a significant proportion of the population, this risk is particularly high. Possible alternatives: (a) use of lower IL-2 doses in combination with IL-7 and IL-15; (b) transduction of TILs with genes encoding constitutive IL-2 production; (c) use of IL-2 mutants with reduced toxicity (e.g., recombinant IL-2 with mutations reducing CD25 binding). However, each of these alternatives has its limitations: low IL-2 doses may be insufficient to support TIL proliferation; genetic modification negates the key advantage of TILs (absence of genetic engineering) and brings their safety profile closer to CAR-T cells; IL-2 mutants require clinical validation.

Conclusion on clinical feasibility: We acknowledge that the proposed strategy is currently conceptual and requires substantial optimisation for clinical application. We do not offer ready-made solutions to all problems but identify them as critical directions for future research. Clinical implementation of the strategy will require stepwise clinical trials with careful monitoring of safety and efficacy.

Thus, TIL therapy in GB is not a mechanical transfer of protocols developed for other diseases but a complex engineering task requiring optimisation of each stage, taking into account the unique features of the microenvironment and CSC biology. TIL therapy is a promising direction, but its introduction into clinical practice for GB will require further optimisation and stepwise clinical trials.

## Conclusion

Glioblastoma remains one of the most aggressive and therapeutically resistant tumours of the central nervous system, and the main reason for this is the ability of CSCs to preserve and transmit adaptive traits acquired under pressure from therapy and the microenvironment. In this review, we have proposed to consider this ability as “tumour memory” — an epigenetically fixed phenotype that ensures survival, recurrence and disease progression. We explicitly acknowledge that this concept is a working hypothesis requiring experimental validation through *in vitro* models of acquired resistance, single-cell chromatin analysis and lineage tracing studies.

The analysis allows us to formulate several key conclusions of fundamental and clinical significance.

First, glioblastoma CSCs and T memory stem cells (TSCM) demonstrate convergent similarity in the use of evolutionarily conserved mechanisms: the WNT/β-catenin signalling pathway, DNA repair systems (including NHEJ and BER) and hierarchical population organisation. However, we critically examined the mechanistic differences in how these pathways function in each cell type (**Table 1**), emphasising that the similarity is heuristic rather than mechanistic. In TSCM, WNT/β-catenin maintains quiescence and self-renewal through TCF1; in CSCs, it stimulates proliferation, invasion and therapy resistance. DNA repair in TSCM serves as the basis for “epigenetic scars” underlying memory maintenance; in CSCs, it is a survival tool against genotoxic stress.

Second, the GB microenvironment represents a highly organised ecosystem that recruits bone marrow-derived cells and forcibly reprograms them into suppressor phenotypes (M2-macrophages, N2-neutrophils, Treg). This process, mediated predominantly by TGFβ and hypoxia, creates a “cold” immune landscape in which effective lymphocytes do not survive in the central tumour zones. The only zone where functionally active lymphocytes may be preserved is the invasive margin — the transitional area between the neoplasm and healthy brain tissue. Here, lymphocytes, although undergoing exhaustion along the TPE → TEX model, may retain a subpopulation of TCF1^+^ precursors capable of proliferation and expansion (**Table 3**). We hypothesise the existence of a therapeutically significant subpopulation and propose to designate it as “invasive margin lymphocytes” (IMLs). We explicitly acknowledge that this is a working hypothesis requiring validation through spatial transcriptomics, multiplex immunofluorescence and single-cell sequencing.

**Table 3:** Comparison of proposed characteristics of IMLs and central TILs in GB.

Feature	IMLs (invasive margin)	Central TILs (hypoxia/necrosis)
Location	Transition zone, contact with healthy tissue	Inside tumour, around necrosis
pO_2_	Moderate hypoxia (closer to normoxia)	Severe hypoxia, anoxia
PD-1	++	+++
TCF1	+	–
CD39	–/low	+++
Proliferative activity	Preserved (TCF1^+^ pool)	Absent
Response to IL−2 ex vivo	Positive (expansion)	Minimal/absent
Therapeutic value	High (substrate for TIL product)	Low

Third, standard cytoreductive therapy (surgery, radiotherapy, temozolomide), while remaining the cornerstone of treatment, does not eliminate the CSC population and may paradoxically enrich the microenvironment with resistant clones. This makes it necessary to combine standard therapy with methods directed against CSCs and their memory. We have proposed a three-stage therapeutic strategy including: 1) precision cytoreduction of the invasive margin with material harvesting for TIL production; 2) “warming up” the microenvironment using cancer vaccines, viral vectors and pharmacological agents (TGFβ inhibitors, CXCR4/CXCL12 antagonists, epigenetic modulators); 3) adoptive transfer of TILs derived from the IML pool, with preservation and enhancement of the TSCM subpopulation for long-term anti-tumour immunity. We acknowledge significant clinical challenges (production time, BBB penetration, IL-2 toxicity) that require further investigation (Section 9.6).

Fourth, the efficacy of the proposed strategy likely depends on the molecular subtype of GB. It holds the greatest promise for the mesenchymal subtype, characterised by the most pronounced immunosuppression and “cold” microenvironment, whereas the proneural subtype, associated with a “warmer” immune landscape, may require less intensive interventions. This underlines the need for a personalised approach that takes into account the molecular-genetic profile of the tumour and the state of the patient’s immune system.

The proposed concept, however, is not without limitations. The convergent similarity between CSCs and TSCM remains predominantly a heuristic analogy requiring experimental validation. The proposed immunophenotype of IMLs is based on extrapolation of data from models of T-cell exhaustion in other cancer types, and direct evidence is still lacking. Furthermore, adaptation of TIL therapy protocols for GB requires solving fundamental technical problems: overcoming the blood-brain barrier, minimising systemic toxicity of lymphodepletion, shortening cell product manufacturing time and optimising culture conditions for TSCM preservation.

Future research (145) should be directed towards: clinical validation of the IML concept using single-cell sequencing and flow cytometry; optimisation of TIL expansion protocols for GB with an emphasis on TSCM preservation; stratification of patients by molecular subtypes for personalised selection of immunomodulation intensity; and development of methods for local delivery of TILs and lymphodepleting agents across the blood-brain barrier using an Ommaya reservoir or other technologies.

Thus, the conducted analysis allows us to conclude that glioblastoma CSCs and T memory stem cells possess convergent similarity, which can serve as a heuristic basis for the development of personalised TIL-based therapy focused on lymphocytes of the invasive margin. Managing tumour memory through the adoptive transfer of TILs derived from the putative IML pool with TSCM preservation represents a new approach to the treatment of glioblastoma, capable of overcoming fundamental resistance mechanisms and ensuring durable disease control. We emphasise that this strategy is currently conceptual and requires substantial preclinical and clinical validation before clinical application.
